# Serum Urate as a Cardiometabolic Risk Enhancer Beyond SCORE2 in Psoriatic Arthritis

**DOI:** 10.3390/jcm15155788

**Published:** 2026-07-24

**Authors:** Lilyan C. Charca, Marta Loredo, Estefanía Pardo, Ignacio Braña, Stefanie Burger, Paula Alvarez, Rubén Queiro

**Affiliations:** 1 Rheumatology Section, Fundació Hospital de l’Esperit Sant, 08923 Santa Coloma de Gramenet, Spain; lily.charca@gmail.com; 2 Rheumatology Division, Central University Hospital of Asturias, 33011 Oviedo, Spain; mloredomart@gmail.com (M.L.); estefaniapardoc@gmail.com (E.P.); i.brana.abascal@hotmail.es (I.B.); stefanie.nam@gmail.com (S.B.); paulavarez992@gmail.com (P.A.); 3 Department of Medicine, School of Medicine, Oviedo University, 33006 Oviedo, Spain; 4 Translational Immunology Division, Health Research Institute of the Principality of Asturias (ISPA), 33011 Oviedo, Spain

**Keywords:** hyperuricemia, urate, cardiometabolic burden, cardiovascular risk, SCORE2, psoriatic arthritis

## Abstract

**Background/Objectives:** Cardiovascular risk (CVR) prediction using SCORE2 may incompletely capture the burden of subclinical atherosclerosis in patients with chronic inflammatory conditions. Identifying simple, accessible markers to refine risk stratification remains an unmet need. This study aimed to evaluate whether serum urate improves detection of subclinical atherosclerosis beyond SCORE2 in a psoriatic arthritis cohort. **Methods:** We conducted a cross-sectional study including 250 patients with psoriatic arthritis fulfilling CASPAR criteria. Vascular assessment comprised carotid and femoral ultrasound and abdominal radiography. Atherosclerotic plaque was defined according to Mannheim criteria. The main outcomes were global plaque (≥1 vascular territory) and extended plaque (≥2 territories). Multivariable logistic regression adjusted for SCORE2 categories assessed independent associations. Incremental value was evaluated using decision curve analysis (DCA), category-free net reclassification improvement (cfNRI), and integrated discrimination improvement (IDI). **Results:** Hyperuricemia prevalence was 21.6%. Patients with hyperuricemia showed a higher prevalence of global plaque (88.9% vs. 62.8%, *p* < 0.001). After adjustment for SCORE2, serum urate was independently associated with global plaque (OR 4.23, 95% CI 1.26–14.2). Notably, 64.3% of patients classified as low–moderate risk already exhibited plaque. In the 50–69-year subgroup, adding serum urate improved reclassification (cfNRI +0.60; IDI +0.031) and was associated with higher net clinical benefit across decision thresholds. The combined model (SCORE2+HU+cIMT) achieved the highest curves, although with limited incremental gain over HU alone. **Conclusions:** SCORE2 categories showed substantial discordance with imaging-defined subclinical atherosclerotic burden in this population. Serum urate, an inexpensive and widely available marker, may help refine cardiovascular risk stratification and identify patients who could benefit from further vascular assessment.

## 1. Introduction

Psoriatic arthritis (PsA) is a chronic immune-mediated inflammatory disease associated with a well-documented excess of cardiovascular (CV) morbidity and mortality. Beyond traditional risk factors, persistent systemic inflammation, metabolic comorbidities, and endothelial dysfunction converge to accelerate atherosclerosis in this population [1]. Patients with PsA frequently exhibit obesity, hypertension, dyslipidemia, insulin resistance, and features of metabolic syndrome, creating a cardiometabolic milieu that differs substantially from that of the general population [1]. Accurate cardiovascular risk (CVR) stratification in PsA therefore remains a major unmet clinical need.

The SCORE2 algorithm, endorsed by the European Society of Cardiology for estimating 10-year CVR in apparently healthy individuals, represents the current standard in Europe [2]. However, SCORE2 was derived from general population cohorts and does not incorporate PsA as a systemic inflammatory disease or disease-specific metabolic abnormality. Consequently, its performance in chronic inflammatory conditions, such as PsA, may be suboptimal. Several studies in psoriatic disease and other immune-mediated inflammatory disorders have shown discordance between calculated SCORE2 categories and imaging-detected subclinical atherosclerosis, suggesting that general-population algorithms may incompletely capture vascular burden in selected subgroups [3,4,5,6,7,8].

Hyperuricemia (HU) is a frequent metabolic abnormality in psoriatic disease. Elevated serum uric acid levels have been linked to obesity, hypertension, insulin resistance, and dyslipidemia, and experimental data suggest mechanistic connections with oxidative stress, endothelial dysfunction, and activation of inflammatory pathways such as the NLRP3 (NOD-, LRR- and pyrin domain-containing protein 3) inflammasome [9,10]. On the other hand, prospective longitudinal evidence from the Spanish CARMA cohort indicates that elevated serum urate levels independently predict cardiovascular events in PsA patients beyond traditional risk scores, reinforcing the clinical relevance of uricemia in CVR stratification [11]. The emerging concept of ‘psout’, a clinical spectrum reflecting the coexistence of HU/gout and PsA, highlights shared pathophysiological mechanisms and clinical phenotypes that may further complicate CVR stratification [9,12].

Imaging-based detection of subclinical atherosclerosis has important prognostic implications. Carotid plaque presence approximately doubles the risk of future CV events in population-based studies, and involvement of multiple vascular territories confers additional risk [13,14]. In this context, risk “enhancers”—factors not included in standard algorithms but capable of refining classification in borderline or intermediate-risk patients—have gained attention. Whether HU could function as such a pragmatic risk enhancer in PsA has not been formally tested.

We hypothesized that HU identifies PsA patients with a greater burden of subclinical atherosclerosis beyond that predicted by SCORE2 and may improve risk stratification, particularly in the clinically challenging 50–69-year subgroup where preventive decisions are most frequently considered. To address this question, we evaluated the association between HU and multisite vascular plaque in a well-characterized PsA cohort and examined its incremental value beyond SCORE2 using multivariable modeling, decision curve analysis, and reclassification metrics.

## 2. Materials and Methods

### 2.1. Study Design and Population

We conducted a cross-sectional observational study including 250 consecutive adult patients fulfilling the CASPAR criteria for PsA [15], attending a tertiary rheumatology center. Patients were enrolled consecutively during routine clinical visits to minimize selection bias. Exclusion criteria included prior clinically overt cardiovascular disease (for SCORE2 applicability analyses), severe renal impairment (eGFR < 30 mL/min/1.73 m^2^), active infection, or incomplete vascular imaging. Patients with prior clinically overt cardiovascular disease were nevertheless retained in the overall cohort for descriptive and exploratory analyses of vascular burden and cardiometabolic phenotype. In contrast, all analyses evaluating SCORE2 performance, reclassification, and incremental predictive value were restricted to SCORE2-eligible patients without established cardiovascular disease.

Demographic data, CVR factors, and PsA-related variables were collected at the time of vascular assessment. Traditional CVR factors included hypertension, diabetes mellitus, dyslipidemia, obesity (BMI ≥ 30 kg/m^2^), and smoking status (current or former). Disease-related variables comprised psoriasis duration, PsA duration, joint phenotype, current biologic therapy, and disease activity measures. Serum uric acid was measured in fasting blood samples using standardized enzymatic methods in the hospital central laboratory. Hyperuricemia was defined as serum uric acid >6.0 mg/dL in women or >7.0 mg/dL in men, consistent with commonly accepted clinical thresholds. A binary variable indicating current urate-lowering therapy was available and included in the definition of HU. This approach was selected to minimize potential misclassification of patients with previously recognized hyperuricemia or gout whose serum urate levels may have normalized under treatment. The study was approved by the Ethics Committee of the Principality of Asturias (protocol 2020.423), and all participants provided written informed consent.

### 2.2. Cardiovascular Risk Assessment

The 2021 European Society of Cardiology guidelines [2] recommend using SCORE2 to estimate ten-year CVR. SCORE2 implementation followed the 2021 ESC prevention guidelines using the moderate cardiovascular risk region calibration applicable to Spain. Patients aged <70 years without diabetes mellitus were evaluated using the standard SCORE2 algorithm, whereas patients aged ≥70 years were classified using SCORE2-OP. Patients with diabetes mellitus were categorized according to the SCORE2-Diabetes framework. Risk categories were assigned using ESC age-specific thresholds: for individuals aged <50 years, low-to-moderate (<2.5%), high (2.5% to <7.5%), and very high (≥7.5%) risk; for individuals aged 50–69 years, low-to-moderate (<5%), high (5% to <10%), and very high (≥10%) risk; and for individuals aged ≥70 years, low-to-moderate (<7.5%), high (7.5% to <15%), and very high (≥15%) risk. Patients with established cardiovascular disease were not included in SCORE2 applicability analyses, in accordance with ESC recommendations.

Analyses focusing on incremental value were prespecified in the 50–69-year subgroup, as this group represents the clinically most actionable range for preventive therapy decisions.

### 2.3. Vascular Imaging

All participants underwent standardized vascular imaging by experienced sonographers blinded to serum urate status. Carotid and femoral arteries were assessed using high-resolution B-mode ultrasound. Atherosclerotic plaque was defined according to Mannheim consensus criteria as a focal structure encroaching into the arterial lumen of at least 0.5 mm or 50% of the surrounding intima–media thickness, or with thickness > 1.5 mm measured from the media–adventitia interface to intima–lumen interface [16]. Carotid intima–media thickness (cIMT) was measured bilaterally in the common carotid artery and averaged. A threshold of >0.9 mm was considered abnormal. Abdominal radiography was performed to detect calcified aortic plaques. Composite vascular outcomes were defined as global plaque: presence of plaque in at least one vascular territory (carotid, femoral, or aortic), or extended plaque in two or more vascular territories. The composite plaque definition was selected to reflect the concept of systemic and multiterritorial atherosclerotic burden in PsA rather than isolated involvement of a single vascular bed.

All vascular ultrasound examinations were performed according to a standardized acquisition protocol by experienced operators blinded to clinical and biochemical data. Reproducibility assessments consistently demonstrated high intraobserver and interobserver agreement for plaque detection and cIMT measurements (ICC for cIMT measurement: 0.92; agreement for plaque detection was also excellent, with kappa coefficient 0.84).

### 2.4. Statistical Analysis

Continuous variables were expressed as mean ± standard deviation (SD) or median with interquartile range (IQR) as appropriate. Categorical variables were expressed as frequencies and percentages. Between-group comparisons (HU vs. non-HU) used Student’s *t*-test or Mann–Whitney U test for continuous variables and chi-square or Fisher’s exact test for categorical variables. To evaluate the independent association between HU and plaque outcomes, multivariable logistic regression models were constructed adjusting for SCORE2 risk category (entered as an ordinal variable) to reflect baseline predicted risk. Additional exploratory sensitivity analyses included adjustment for sex in multivariable models evaluating the association between HU and plaque outcomes. Sex-stratified analyses were also explored; however, interpretation was considered exploratory because of the limited number of women with HU. Detailed exposure data regarding concomitant medications potentially influencing serum urate levels or cardiovascular risk (including diuretics, antihypertensive agents, statins, NSAIDs, corticosteroids, and urate-lowering therapies) were not systematically available in the analytic dataset and therefore were not incorporated into multivariable models. Collinearity was assessed using variance inflation factors (VIFs), and model discrimination was evaluated using area under the receiver operating characteristic curve (AUC). Incremental predictive value beyond SCORE2 was assessed using category-free Net Reclassification Improvement (cfNRI), quantifying correct upward reclassification among patients with plaque and correct downward reclassification among those without plaque; Integrated Discrimination Improvement (IDI), measuring change in mean predicted probabilities between events and non-events; and Categorical NRI, calculated using predefined 10%, 20%, and 30% risk thresholds. These metrics were selected because high plaque prevalence limits the interpretability of AUC alone in this setting. Clinical utility was evaluated using Decision Curve Analysis (DCA), which estimates net clinical benefit across a range of threshold probabilities representing potential treatment decision cut-offs (10–30%). Models compared included: SCORE2 alone, SCORE2+HU, SCORE2+cIMT, and SCORE2+HU+cIMT. Net benefit curves were interpreted relative to “treat-all” and “treat-none” strategies. Threshold probabilities in the DCA were interpreted as conceptual thresholds for considering intensified cardiovascular evaluation or preventive management rather than fixed treatment initiation cut-offs.

All analyses were performed using Python 3.11 (pandas, statsmodels, scikit-learn) and R 4.3 (rmda package for DCA). Two-sided *p*-values < 0.05 were considered statistically significant.

## 3. Results

### 3.1. Study Population

A total of 250 patients with PsA were included. Median age was 63.2 years (IQR 52.6–69.6), and 119 (47.6%) were women. Median psoriasis and PsA duration were 24.0 and 11.0 years, respectively. Traditional CVR factors were frequent: hypertension in 35.6%, dyslipidemia in 67.6%, obesity in 22.4%, diabetes in 10.0%, and ever-smokers at 50.8%. Overall, 70.8% were receiving biologic therapy at the time of vascular evaluation. Table 1 reflects the overall study cohort, including a small number of patients with prior cardiovascular disease who were retained for descriptive characterization of vascular burden and cardiometabolic phenotype.

Hyperuricemia prevalence was 21.6% (n = 54), with marked sex disparity (32.8% in men vs. 9.2% in women). Hyperuricemia patients had significantly higher BMI (29.2 ± 4.1 vs. 27.3 ± 3.8 kg/m^2^, *p* = 0.02), higher systolic blood pressure (127.8 ± 12.0 vs. 122.1 ± 11.4 mmHg, *p* = 0.03), and lower HDL cholesterol (48.3 ± 12.5 vs. 57.0 ± 13.2 mg/dL, *p* = 0.01) compared with non-HU individuals. Other traditional risk factors did not differ significantly. A detailed comparison between HU and non-HU patients is shown in Supplementary Table S1.

### 3.2. Association Between Hyperuricemia and Plaque Burden

Plaque prevalence was high across the cohort but consistently greater among HU patients. Global plaque was present in 88.9% of HU versus 62.8% of non-HU patients (*p* < 0.001). Similar patterns were observed across vascular territories: carotid plaque (53.7% vs. 31.1%, *p* = 0.004), femoral plaque (85.2% vs. 56.6%, *p* < 0.001), aortic plaque (55.6% vs. 25.0%, *p* < 0.001), and extended plaque (64.8% vs. 37.2%, *p* = 0.001).

In multivariable logistic regression adjusting for SCORE2 categories, HU was independently associated with global plaque (OR 4.23, 95% CI 1.26–14.2, *p* = 0.02) and aortic plaque (OR 2.60, 95% CI 1.20–5.62, *p* = 0.015). Associations with femoral and extended plaque showed similar directionality but did not reach statistical significance (Table 2).

In a sensitivity analysis adjusting for DAPSA and traditional CVR factors, HU remained independently associated with global plaque (aOR 2.80, 95% CI 1.02–7.64; *p* < 0.05) (Table 3). Although DAPSA was associated with plaque in univariable analysis, its effect attenuated after accounting for cardiometabolic factors, whereas HU retained independent significance.

In sex-adjusted sensitivity analyses, the association between HU and plaque outcomes remained directionally consistent. After adjustment for SCORE2 category and sex, HU was associated with higher odds of global plaque (OR 2.52, 95% CI 0.87–7.32), femoral plaque (OR 2.22, 95% CI 0.87–5.65), aortic plaque (OR 2.62, 95% CI 1.22–5.63), and extended plaque (OR 1.74, 95% CI 0.79–3.85), although only the association with aortic plaque remained statistically significant.

In an exploratory sensitivity analysis restricted to ultrasound-detected plaque, defined as carotid and/or femoral plaque, the direction of the association between HU and plaque burden remained consistent with the primary composite outcome, although the association was attenuated and did not reach statistical significance after adjustment for SCORE2 category (OR 1.83, 95% CI 0.72–4.65; *p* = 0.203). Combined carotid–femoral ultrasound plaque was observed in 53.7% of HU patients versus 26.0% of non-HU individuals (*p* = 0.001). After adjustment for SCORE2 category, HU remained directionally associated with combined carotid–femoral plaque burden (OR 2.02, 95% CI 1.01–4.07; *p* = 0.045).

### 3.3. Plaque Prevalence Across SCORE2 Categories

**Figure 1:**
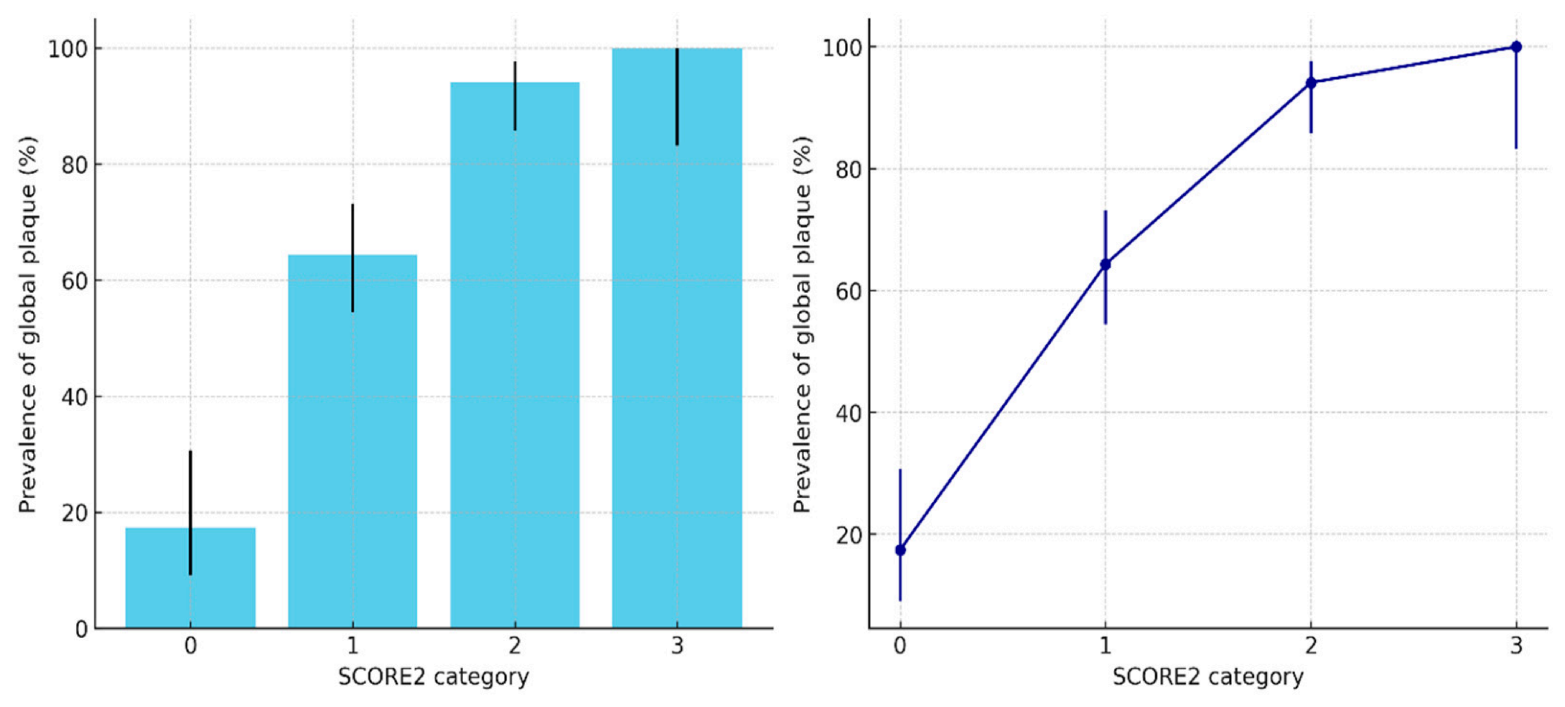
Prevalence of global plaque by SCORE2 category. Prevalence of global atherosclerotic plaque across SCORE2 categories in patients with psoriatic arthritis. (**Left**) panel: bar representation with Wilson 95% confidence intervals (CIs). (**Right**) panel: line representation with Wilson 95% CIs. A stepwise increase was observed between categories 0, 1, and 2, with a ceiling effect between categories 2 and 3. In category 3, the upper 95% CI bound is truncated at 100%, resulting in a one-sided interval.

Global plaque prevalence increased stepwise across SCORE2 categories (*p* < 0.001): 17.4% in category 0 (low risk), 64.3% in category 1 (low–moderate), 94.1% in category 2 (high), and 100% in category 3 (very high). Pairwise comparisons showed significant differences between categories 0 and 1 and between 1 and 2 (both *p* < 0.001), whereas no difference was observed between categories 2 and 3 (*p* = 0.57), indicating a ceiling effect at higher risk strata (Figure 1).

Notably, 64.3% of patients classified as low–moderate risk already had plaque, highlighting substantial discordance between algorithm-estimated risk and structural vascular burden (Table 4).

### 3.4. Incremental Value in the 50–69-Year Subgroup

In the prespecified 50–69-year subgroup, HU further stratified plaque risk within each SCORE2 category. Addition of HU to SCORE2 improved reclassification, with a category-free net reclassification improvement (cfNRI) of +0.57 and integrated discrimination improvement (IDI) of +0.046. The observed cfNRI improvement was driven predominantly by correct downward reclassification among patients without plaque, whereas the event component was negative. Accordingly, the incremental value of HU appeared to derive mainly from improved identification of individuals less likely to harbor plaque rather than enhanced sensitivity for plaque-positive cases.

In contrast, adding cIMT >0.9 mm to SCORE2 yielded limited incremental discrimination (IDI ≈ 0; cfNRI +0.10), with a negative event component and non-significant reclassification metrics. Combining HU and cIMT preserved the incremental benefit attributable to HU, without evidence of meaningful additional improvement beyond the HU-containing model alone (Table 5).

**Figure 2:**
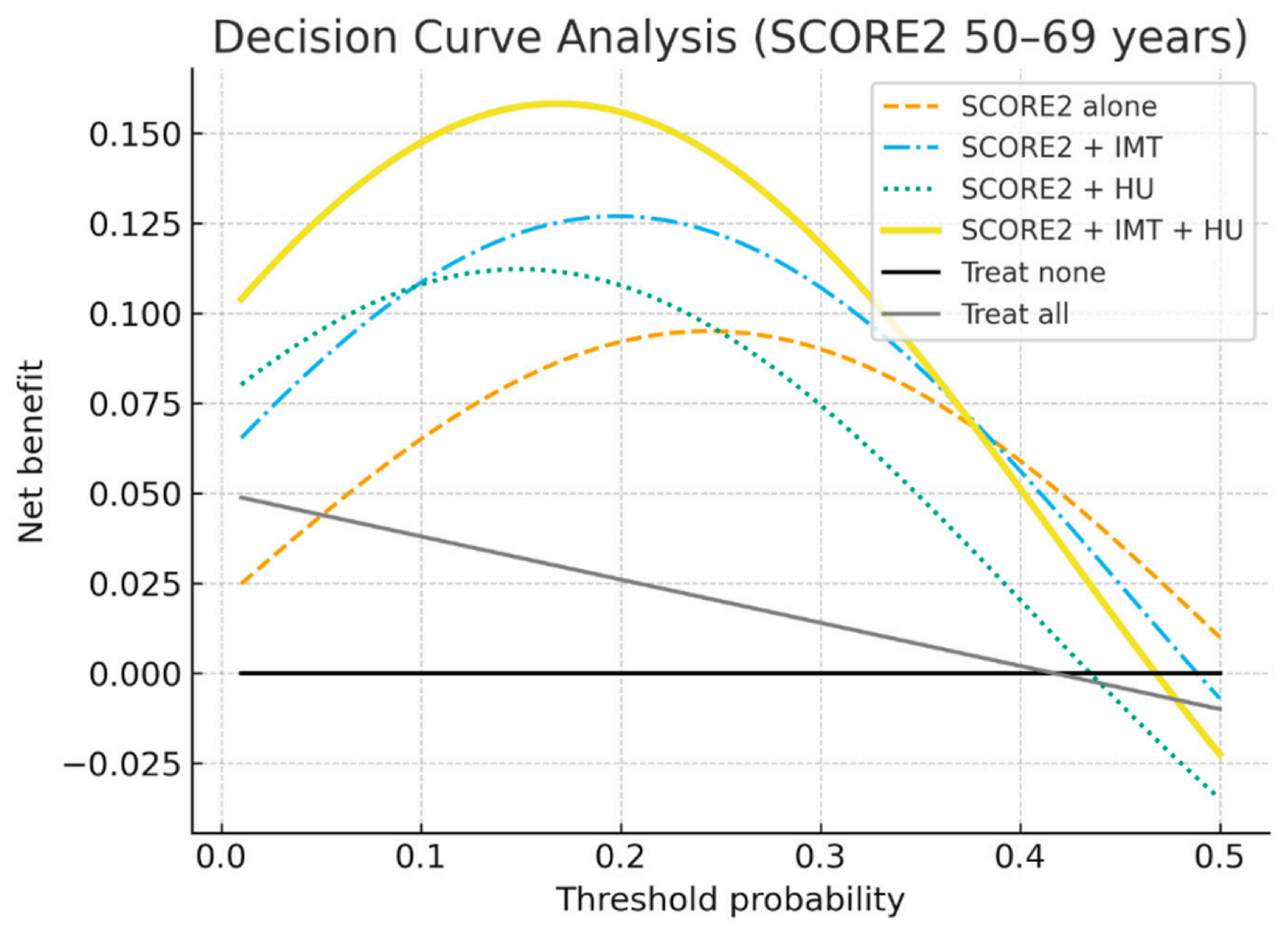
Decision curve analysis in the 50–69 age group. Decision curve analysis evaluating the net clinical benefit of different models for detecting global atherosclerotic plaque in patients with psoriatic arthritis aged 50–69 years. The dashed orange line represents SCORE2 alone; the blue dash-dotted line, SCORE2 plus carotid intima–media thickness (cIMT); the green dotted line, SCORE2 plus hyperuricemia (HU); and the yellow solid line, SCORE2 plus IMT and HU combined. The black horizontal line corresponds to the strategy of treating no patients, and the gray line to treating all patients. Threshold probabilities are described as conceptual points for further cardiovascular evaluation or preventive management rather than fixed treatment-initiation cut-offs.

Decision curve analysis demonstrated that models incorporating HU provided higher net clinical benefit than SCORE2 alone or SCORE2 combined with cIMT across clinically relevant threshold probabilities (10–30%). The combined model (SCORE2+HU+cIMT) yielded the highest net benefit curves; however, the incremental gain over SCORE2+HU alone was minimal, indicating that most of the clinical utility was driven by HU rather than by cIMT (Figure 2). However, categorical NRI using predefined 10–20–30% thresholds remained null across all models, indicating that the observed improvements in continuous reclassification did not translate into substantial shifts across conventional SCORE2-based clinical decision categories.

## 4. Discussion

This study provides clinically meaningful evidence that HU identifies a subgroup of patients with PsA who harbor a disproportionately high burden of subclinical atherosclerosis beyond that estimated by SCORE2. Our findings contribute to the ongoing debate regarding the performance of general-population cardiovascular risk algorithms in immune-mediated inflammatory diseases and suggest that serum urate may function as a pragmatic risk enhancer in PsA. Three principal observations support this interpretation: (1) HU was clustered with adverse metabolic traits and marked structural vascular involvement; (2) HU was independently associated with global and aortic plaque after adjustment for SCORE2 categories; and (3) addition of HU improved reclassification and clinical net benefit in the clinically actionable 50–69-year subgroup, whereas cIMT yielded limited incremental value.

Psoriatic arthritis is characterized not only by musculoskeletal inflammation but also by a complex cardiometabolic phenotype in which systemic inflammation, adiposity, insulin resistance, and dyslipidemia interact [1,3,17]. HU appears to occupy a central position within this network [18]. In our cohort, HU prevalence reached 21.6%, consistent with prior reports in psoriatic disease [9,12]. The marked sex disparity (higher in men) mirrors known biological differences in urate handling and hormonal modulation of urate renal excretion [19]. Importantly, HU did not occur in isolation. Patients with elevated uric acid levels exhibited higher body mass index, elevated systolic blood pressure, and lower HDL cholesterol, reinforcing its integration within a pro-atherogenic metabolic profile. This clustering raises the question of whether HU is merely a surrogate of metabolic burden or whether it contributes independently to vascular pathology. Experimental and translational data support biological plausibility for a contributory role. Uric acid has been implicated in endothelial dysfunction through reduced nitric oxide bioavailability, induction of oxidative stress, activation of the renin–angiotensin system, and stimulation of inflammatory pathways including NLRP3 inflammasome activation [9,10]. In PsA, where systemic inflammation is persistent and often accompanied by adipose tissue-derived cytokines, urate-mediated endothelial injury may synergize with immune activation to accelerate vascular remodeling. While our cross-sectional design does not permit causal inference, the persistence of associations after adjustment for SCORE2 suggests that HU captures vascular risk not fully encompassed by traditional variables.

A major insight from this study is the discordance between algorithm-estimated CVR and structural vascular damage detected by imaging. Although plaque prevalence increased stepwise across SCORE2 categories, a striking 64.3% of patients classified as low–moderate risk already had detectable plaque. This finding supports the concept that general-population algorithms may incompletely reflect imaging-defined subclinical atherosclerotic burden in PsA [6]. Such discordance has been reported in other immune-mediated inflammatory diseases where inflammation-related mechanisms accelerate vascular aging independently of classical risk factors [7,8]. Our data extend this concept to PsA in the context of SCORE2, the current European standard.

Interestingly, at higher SCORE2 categories, plaque prevalence approached universality, resulting in a ceiling effect. This phenomenon illustrates a broader methodological challenge: when outcome prevalence is high, discrimination metrics such as AUC lose interpretability. AUC values near 0.50 in this context do not necessarily reflect absence of risk gradient but rather limited separation between events and non-events when most individuals harbor structural disease. For this reason, we emphasized prevalence gradients, reclassification indices, and decision curve analysis, which better capture clinical utility under conditions of high baseline prevalence. Additionally, odds ratios may overestimate the apparent magnitude of association, and therefore effect estimates should be interpreted primarily in terms of directionality and consistency across complementary analyses rather than as precise measures of relative prevalence.

Demonstrating association alone is insufficient to justify clinical integration. Therefore, we examined whether HU adds incremental predictive information beyond SCORE2. In the 50–69-year subgroup—where preventive strategies such as statin initiation are most debated—adding HU improved category-free net reclassification improvement (cfNRI) and integrated discrimination improvement (IDI). Importantly, the cfNRI improvement was largely attributable to downward reclassification among non-events, whereas the event component was negative. This pattern suggests that hyperuricemia may be more informative for refining identification of lower-plaque individuals within intermediate SCORE2 strata than for improving detection of plaque-positive patients. The absence of meaningful categorical NRI improvement further supports cautious interpretation of the incremental clinical utility. In contrast, addition of cIMT >0.9 mm yielded minimal incremental discrimination. This is noteworthy because cIMT has historically been considered a subclinical marker of atherosclerosis [13,14]. Importantly, despite improvements in category-free reclassification metrics, categorical NRI remained null across models. This finding suggests that the incremental value of HU may primarily refine continuous risk estimation rather than substantially alter threshold-based clinical decision-making using conventional SCORE2 categories.

Our findings align with the evolving concept that plaque detection carries greater prognostic relevance than cIMT alone [13,14]. In this context, the limited incremental contribution observed for cIMT thresholding in our models should not be interpreted as evidence against vascular imaging, but rather as suggesting that serum urate may provide complementary metabolic information beyond SCORE2 categories within this specific analytical framework.

The DCA findings further support the role of HU as a clinically relevant risk enhancer. Although the combined model (SCORE2+HU+cIMT) achieved the highest net benefit across decision thresholds, the additional contribution of cIMT beyond HU was marginal. This pattern suggests that the improvement in clinical utility is primarily attributable to HU, while cIMT adds limited incremental value in this context. Importantly, the DCA was not intended to define rigid treatment thresholds. Rather, the observed net benefit should be interpreted as reflecting potential utility for identifying patients who may warrant further cardiovascular evaluation, closer cardiometabolic surveillance, or consideration of more individualized preventive strategies within clinically uncertain SCORE2 strata. Additionally, we do not propose serum urate as a substitute for vascular imaging. Instead, urate measurement may represent a pragmatic adjunctive marker capable of identifying patients who could benefit from more detailed vascular assessment.

From a practical standpoint, our results suggest that serum uric acid measurement—inexpensive, widely available, and routinely obtained—may assist in refining CVR discussions in PsA. This is particularly relevant in patients falling into borderline or low–moderate SCORE2 strata, where clinicians face uncertainty regarding initiation of preventive therapies. Importantly, we do not propose that HU replace imaging or established algorithms. Rather, HU may serve as an early signal prompting further evaluation. In resource-limited settings where vascular ultrasound is not readily available, uric acid may offer a pragmatic intermediate step in risk refinement.

Therapeutic implications warrant cautious interpretation. Randomized trials of urate-lowering therapy (ULT) in the general population have yielded mixed cardiovascular results [20,21,22]. However, PsA represents a distinct pathophysiological environment in which urate metabolism intersects with systemic inflammation. Whether lowering serum urate translates into reduced vascular progression or fewer cardiovascular events in PsA remains unknown. Accordingly, our findings should not be interpreted as evidence supporting urate-lowering therapy for cardiovascular prevention. Rather, hyperuricemia may represent a clinically accessible marker of adverse cardiometabolic phenotype requiring closer cardiovascular assessment and risk-factor optimization [23]. Our findings support considering HU not merely as a biochemical curiosity but as a potential modifier of cardiometabolic phenotype.

Previous imaging-based studies in PsA have demonstrated high rates of carotid plaque and frequent reclassification when imaging is incorporated into risk assessment [6]. However, most focused on structural detection rather than metabolic enhancers. Studies examining uric acid in psoriatic disease have reported associations with metabolic syndrome and vascular markers, but formal evaluation of incremental value beyond contemporary European algorithms using decision-analytic frameworks has been limited. By integrating multisite plaque assessment, formal reclassification metrics, and DCA, our study provides a more comprehensive evaluation of HU as a risk enhancer. The use of composite global and extended plaque outcomes acknowledges that atherosclerosis is systemic rather than confined to a single vascular bed.

This study benefits from a well-characterized PsA cohort, standardized vascular imaging across multiple arterial territories, and application of advanced analytic methods beyond simple association testing. The prespecified focus on the 50–69-year subgroup enhances relevance to real-world clinical decision-making. Notably, the association between HU and plaque remained significant after adjustment for disease activity and classical CVR factors, whereas the effect of DAPSA attenuated in the multivariable model. This pattern indicates that urate levels may capture vascular risk dimensions not fully accounted for by inflammatory burden alone, reinforcing its potential role as a metabolic risk enhancer in PsA.

Nevertheless, limitations must be acknowledged. The cross-sectional design precludes determination of temporal sequence between HU and plaque development. Residual confounding cannot be excluded, particularly given complex interactions between inflammation, adiposity, renal function, and medication exposure. Individual renal function parameters and detailed pharmacologic exposure data were not systematically available, precluding dedicated analyses evaluating the potential influence of kidney function or concomitant therapies on serum urate levels and vascular outcomes. HU was measured at a single time point, and cumulative exposure was not assessed. Detailed information regarding gout diagnosis and urate-lowering therapy exposure was not systematically available, precluding dedicated treatment-specific sensitivity analyses. The fully adjusted multivariable model also showed evidence of instability, reflected by wide confidence intervals and unexpected coefficient directions for some cardiometabolic variables, likely related to collinearity and the high prevalence of plaque outcomes in this cohort. Additionally, plaque is a surrogate marker, albeit one with recognized prognostic significance. External validation in independent cohorts is essential before widespread adoption. Recent data from the CARMA cohort appear to clearly reinforce the role of HU as a CVR enhancer in psoriatic disease [11]. Moreover, development of PsA-specific risk algorithms incorporating inflammatory and metabolic variables may ultimately be required to address systematic underestimation inherent to general-population tools.

Although the CARMA cohort offers some insight [11], additional prospective studies are required to assess whether HU independently predicts cardiovascular events in PsA. Integration of inflammatory biomarkers, imaging findings, and metabolic markers into composite risk models may offer improved discrimination. Mechanistic investigations exploring the interplay between urate metabolism, adipokines, and immune activation could clarify whether HU is a mediator or marker of vascular risk. Ultimately, refinement of CVR assessment in PsA will likely require disease-adapted frameworks. Our findings position HU as a candidate component of such models.

## 5. Conclusions

In this cohort of patients with PsA, HU was common and independently associated with multiterritorial subclinical atherosclerosis beyond SCORE2 classification. A substantial proportion of individuals categorized as low–moderate CVR already exhibited structural plaque, underscoring discordance between general-population risk categories and imaging-defined vascular burden. Hyperuricemia improved risk reclassification and net clinical benefit in the clinically actionable 50–69-year subgroup, whereas cIMT provided limited incremental value. These findings support considering serum uric acid as a pragmatic CVR enhancer in PsA. Prospective studies with hard CV endpoints are required to determine whether incorporation of HU into disease-adapted risk models improves outcome prediction and preventive strategies.

## Figures and Tables

**Table 1:** Disease features of the study population and sex-related differences.

Disease Feature	Total n: 250	Men n: 131	Women n: 119	*p*-Value
Age, yrs, median (IQR)	63.2 (52.6–69.6)	62.6 (52.6–71.5)	63.9 (52.7–69.1)	NS
Psoriasis duration, yrs, median (IQR)	24.0 (14.0–39.5)	23.0 (12.0–38.0)	25.0 (14.0–41.5)	NS
PsA duration, yrs, median (IQR)	11.0 (7.0–13.0)	10.0 (7.0–13.5)	11.0 (8.0–13.0)	NS
BMI, median (IQR)	27.2 (25.0–29.7)	27.8 (25.5–29.7)	26.7 (24.0–29.6)	NS
Systolic blood pressure, mmHg, median (IQR)	123.0 (117.0–130.0)	125.0 (120.0–130.0)	120.0 [115.0–127.0)	0.001
Cholesterol mg/dL, median (IQR)	195.0 (168.0–227.5)	191.0 (164.5–222.5)	200.0 [170.0–234.5)	NS
HDL-cholesterol, mg/dL, median (IQR)	54.5 (46.0–63.0)	51.00 (42.0–61.0)	57.0 (51.0–66.0)	<0.001
HAQ, median (IQR)	0.75 (0.25–1.12)	0.62 (0.00–1.06)	0.87 (0.50–1.12)	0.006
PSAID12, median (IQR)	3.73 (1.21–5.24)	3.50 (1.07–5.40)	3.95 (2.03–4.93)	NS
cIMT, mm, median (IQR)	0.76 (0.67–0.90)	0.78 (0.68–0.95)	0.76 (0.65–0.90)	NS
Psoriasis family history, n (%)	123 (49.2)	71 (54.2)	52 (43.7)	NS
Oligoarthritis, n (%)	115 (46.0)	59 (45.0)	56 (47.1)	NS
Mixed, n (%)	80 (32.0)	55 (42.0)	25 (21.0)	<0.001
Polyarthritis, n (%)	47 (18.8)	15 (11.5)	32 (26.9)	<0.001
Axial, n (%)	8 (3.2)	2 (1.5)	6 (5.0)	NS
DIP disease, n (%)	37 (14.8)	21 (16.0)	16 (13.4)	NS
Dactylitis, n (%)	93 (37.2)	43 (32.8)	50 (42.0)	NS
Enthesitis, n (%)	83 (33.2)	53 (40.5)	30 (25.2)	0.015
IBD, n (%)	9 (3.6)	5 (3.8)	4 (3.4)	NS
Uveitis, n (%)	8 (3.2)	7 (5.3)	1 (0.8)	NS
Former smokers, n (%)	73 (29.2)	46 (35.1)	27 (22.7)	NS
Current smokers, n (%)	54 (21.6)	27 (20.6)	27 (22.7)	NS
CVD, n (%)	12 (4.8)	9 (6.9)	3 (2.5)	NS
Erosive disease, n (%)	55 (22.0)	30 (22.9)	25 (21.0)	NS
Mild BSA (≤3%), n (%)	112 (44.8)	59 (45.0)	53 (44.5)	NS
Moderate–severe BSA (>3%), n (%)	15 (6.0)	13(9.9)	2 (1.7%)	0.044
Nail disease, n (%)	123 (49.2)	65 (49.6)	58 (48.7)	NS
Biologics, n (%)	177 (70.8)	89 (67.9)	88 (73.9)	NS
Rem-low DAPSA, n (%)	172 (68.8)	101 (77.1)	71 (59.7)	0.007
Moderate–high DAPSA, n (%)	76 (30.4)	28 (21.4%)	48 (40.3)	0.007
MDA, n (%)	105 (42.0)	62 (47.3)	43 (36.1)	NS
SCORE2 < 50 low, n (%)	46 (18.4)	16 (12.2)	30 (25.2)	<0.001
SCORE2 < 50 moderate, n (%)	15 (6.0)	15 (11.5)	0 (0.0)	<0.001
SCORE2 < 50 very high, n (%)	2 (0.8)	2 (1.5)	0 (0.0)	NS
SCORE2 50–69 low–mod, n (%)	75 (30.0)	28 (21.4)	47 (39.5)	<0.001
SCORE2 50–69 high, n (%)	47 (18.8)	36 (27.5)	11 (9.2)	<0.001
SCORE2 50–69 very high, n (%)	9 (3.6)	6 (4.6)	3 (2.5)	NS
SCORE2 OP moderate, n (%)	8 (3.2)	3 (2.3)	5 (4.2)	NS
SCORE2 OP high, n (%)	21 (8.4)	14 (10.7)	7 (5.9)	0.028
SCORE2 OP very high, n (%)	8 (3.2)	8 (6.1)	0 (0.0)	0.028
SCORE2 DM high, n (%)	7 (2.8)	3 (2.3)	4 (3.4)	NS
SCORE2 DM very high, n (%)	12 (4.8)	0 (0.0)	12 (10.1)	NS
Depression, n (%)	46 (18.4)	15 (11.5)	31 (26.1)	0.005
Fibromyalgia, n (%)	28 (11.2)	6 (4.6)	22 (18.5)	0.001
Carotid plaque, n (%)	90 (36.0)	49 (37.4)	41 (34.5)	NS
Femoral plaque, n (%)	157 (62.8)	95 (72.5)	62 (52.1)	0.001
Aortic calcification, n (%)	79 (31.6)	44 (33.6)	35 (29.4)	NS
Obesity, n (%)	56 (22.4)	25 (19.1)	31 (26.1)	NS
Hypertension, n (%)	89 (35.6)	52 (39.7)	37 (31.1)	NS
Diabetes, n (%)	25 (10.0)	9 (6.9)	16 (13.4)	NS
Dyslipidemia, n (%)	169 (67.6)	91 (69.5)	78 (65.5)	NS
Hyperuricemia, n (%)	54 (21.6)	43 (32.8)	11 (9.2)	<0.001

Yrs: years; IQR: interquartile range; PsA: psoriatic arthritis; BMI: body mass index; HDL: high-density lipoprotein; HAQ: health assessment questionnaire; PsAID: PsA impact of disease; cIMT: carotid intima–media thickness; mm: millimeter; DIP: distal interphalangeal joint; IBD: inflammatory bowel disease; CVD: cardiovascular disease; BSA: body surface area; rem: remission; DAPSA: Disease Activity Index for PSoriatic Arthritis; MDA: minimal disease activity; SCORE2: systematic coronary risk evaluation 2; OP: older-people; DM: diabetes mellitus; n: number; NS: non-significant. SCORE2 categories shown in Table 1 reflect the age-specific ESC framework, including SCORE2 for patients aged <70 years without diabetes, SCORE2-OP for older individuals, and SCORE2-Diabetes for patients with diabetes mellitus.

**Table 2:** Adjusted logistic regression for hyperuricemia predicting plaque beyond SCORE2.

Outcome	OR	95% CI	*p* Value
Global plaque	4.23	1.26–14.2	0.02
Carotid plaque	1.75	0.87–3.52	0.11
Femoral plaque	2.11	0.93–4.78	0.07
Aortic plaque	2.60	1.20–5.62	0.015
Extended plaque	2.05	0.97–4.32	0.06

OR: odds ratios; CI: confidence interval.

**Table 3:** Fully adjusted logistic regression model for global plaque.

Variable	Adjusted OR	95% CI	*p* Value
Hyperuricemia	2.80	1.02–7.64	0.045
DAPSA	1.40	0.93–2.11	0.110
Hypertension	10.82	0.98–118.90	0.052
Dyslipidemia	4.09	2.10–7.96	<0.001
Smoking	1.87	0.37–9.36	0.448
Obesity (BMI ≥ 30 kg/m^2^)	0.18	0.02–1.72	0.136
Diabetes mellitus	6.49	2.76–15.29	<0.001

The outcome was the presence of global atherosclerotic plaque (carotid, femoral, or aortic). Multicollinearity was assessed using variance inflation factors (all VIF < 1.2). Internal stability of the fully adjusted model was examined using non-parametric bootstrap resampling (400 replicates), yielding consistent percentile 95% confidence intervals for the principal predictors (hypertension, dyslipidemia, hyperuricemia, and smoking). The fully adjusted model should be interpreted as exploratory because the high prevalence of plaque and the relatively limited number of non-events may reduce coefficient stability when multiple correlated cardiometabolic variables are entered simultaneously.

**Table 4:** Prevalence of plaque outcomes by SCORE2 category in psoriatic arthritis.

SCORE2 Category	Global Plaque % (95% CI)	Carotid % (95% CI)	Femoral % (95% CI)	Aortic % (95% CI)	Extended % (95% CI)
**0** (<50 y, low), n: 46	17.4% (9.1–30.7)	8.7% (3.2–21.0)	13.0% (6.1–25.6)	4.3% (1.2–14.5)	0% (0.0–7.6)
**1** (50–69 y, low–moderate), n: 98	64.3% (54.4–73.1)	33.7% (25.3–43.4)	49.0% (39.6–58.4)	18.4% (12.0–27.2)	33.7% (25.3–43.4)
**2** (50–69 or ≥70 y, high), n: 68	94.1% (85.8–97.7)	60.3% (47.9–71.5)	82.4% (71.1–89.8)	41.2% (29.9–53.7)	73.5% (61.4–82.9)
**3** (very high risk/prior CV event), n: 19	100% (83.2–100)	73.7% (51.0–88.6)	84.2% (62.4–94.5)	57.9% (36.3–76.9)	78.9% (56.7–91.4)

Values represent the percentage of patients with each plaque outcome in the corresponding SCORE2 category, with Wilson 95% confidence intervals (CIs). Global plaque = any positive territory (carotid, femoral, or aortic); extended plaque = ≥2 vascular territories. SCORE2 categories were defined according to ESC 2021 guidelines: <50 years (0 = low; 1 = moderate; 2 = high; 3 = very high if prior CV event), 50–69 years (1 = low–moderate, 2 = high, 3 = very high), ≥70 years (1 = moderate, 2 = high, 3 = very high).

**Table 5:** Reclassification metrics for SCORE2 models in the 50–69-year subgroup.

Model Comparison	cfNRI Total (95% CI)	*p* Value	cfNRI (Events/Non-Events)	IDI (95% CI)	*p* Value	Categorical NRI
SCORE2 vs. +cIMT	+0.101 (−0.160 to +0.433)	0.446	−0.485/+0.586	−0.0001 (−0.0007 to +0.044)	0.380	0.00
SCORE2 vs. +HU	+0.565 (+0.333 to +0.784)	0.006	−0.366/+0.931	+0.046 (+0.005 to +0.104)	0.008	0.00
SCORE2 vs. +HU+cIMT	+0.565 (+0.077 to +0.831)	0.030	−0.366/+0.931	+0.046 (+0.008 to +0.119)	0.006	0.00

Compared with SCORE2 alone, addition of hyperuricemia improved category-free net reclassification improvement (cfNRI) and integrated discrimination improvement (IDI) in the 50–69-year subgroup. However, the cfNRI improvement was driven predominantly by downward reclassification among patients without plaque, whereas the event component remained negative. Accordingly, the incremental value of hyperuricemia appeared to derive mainly from improved identification of individuals who are less likely to harbor plaque rather than enhanced sensitivity for plaque-positive cases. Addition of cIMT alone did not materially improve discrimination. Confidence intervals and *p* values were estimated using bootstrap resampling. Category-based NRI using 10–20–30% thresholds remained null across models, likely reflecting both the categorical structure of SCORE2 and the high prevalence of plaque in this cohort.

## Data Availability

The data presented in this study are available on request from the corresponding author. Due to patient confidentiality and institutional policies, raw individual-level data cannot be made publicly available.

## Supplementary Materials

The following supporting information can be downloaded at https://www.mdpi.com/article/10.3390/jcm15155788/s1. Table S1: Exploratory comparison between hyperuricemic and non-hyperuricemic patients.
