# Supplementary material for: Serum Urate as a Cardiometabolic Risk Enhancer Beyond SCORE2 in Psoriatic Arthritis
Source: J Clin Med. 2026 Jul 24;15(15):5788. doi: 10.3390/jcm15155788 (PMC13467134; doi:10.3390/jcm15155788)
Supplement: Supplementary file 1 [file jcm-15-05788-s001.zip › jcm-4268934-supplementary.pdf]

Table S1. Exploratory comparison between hyperuricemic and non-hyperuricemic patients

Patients were classified as hyperuricemic (HU) when the cardiovascular risk factor coding field included the value 5, corresponding to hyperuricemia. Patients could simultaneously present multiple cardiovascular risk factors.

Sociodemographic profile

Variable	HU	Non-HU	p-value
Women sex	11/54 (20.4%)	108/196 (55.1%)	0.000
BMI	28.7 (25.8-31.9)	27.0 (24.6-29.4)	0.007
Smoking	11/54 (20.4%)	43/196 (21.9%)	0.854

Clinical profile

Variable	HU	Non-HU	p-value
DAPSA	1.0 (1.0-2.0)	1.0 (0.0-2.0)	0.333
HAQ	0.9 (0.4-1.2)	0.6 (0.2-1.0)	0.041
PsAID12	3.8 (1.9-5.4)	3.6 (1.2-5.0)	0.404
Dactylitis	15/54 (27.8%)	78/196 (39.8%)	0.114
Enthesitis	23/54 (42.6%)	60/196 (30.6%)	0.105
Erosive disease	14/54 (25.9%)	41/196 (20.9%)	0.460
MDA	20/54 (37.0%)	85/196 (43.4%)	0.439

Cardiometabolic profile and vascular outcomes

Variable	HU	Non-HU	p-value
Systolic BP	127.0 (120.5-134.8)	122.0 (115.0-127.0)	0.000
HDL cholesterol	49.5 (39.8-55.0)	56.0 (47.8-65.0)	0.000
Carotid plaque	29/54 (53.7%)	61/196 (31.1%)	0.004
Femoral plaque	46/54 (85.2%)	111/196 (56.6%)	0.000
Aortic plaque	30/54 (55.6%)	49/196 (25.0%)	0.000

Therapeutic profile

Variable	HU	Non-HU	p-value
Biologic therapy	9/54 (16.7%)	52/196 (26.5%)	0.155

Interpretative summary

Hyperuricemic patients exhibited a predominantly male cardiometabolic phenotype, with trends toward higher BMI and lower HDL cholesterol. Vascular burden was consistently greater among HU patients, particularly regarding femoral and aortic plaque involvement. Clinical disease activity measures such as DAPSA, HAQ, and PsAID12 did not show major differences between groups, supporting the interpretation that hyperuricemia may capture a predominantly cardiometabolic rather than inflammatory vascular-risk signal. Therapeutic exposure to biologics was broadly similar between groups.
